# Systemic Sarcoidosis With Neurosarcoidosis and Obstructive Hydrocephalus: A Radiologic–Pathologic Case Report

**DOI:** 10.14740/jmc5337

**Published:** 2026-08-21

**Authors:** Talgat Muminov, Irina Sergeeva, Gulnar Mambetova, Zhamilya Zholdybay, Anastasiya Goncharova, Anar Rakisheva, Gulstan Yessetova, Galiya Akhmetova, Dias Tolesbayev, Gaukhar Aitbay, Abylay Mels, Yevgeniya Filippenko

**Affiliations:** a Department of Pulmonology, Asfendiyarov Kazakh National Medical University, Almaty, Kazakhstan; b Almaty City Pathology Bureau, Almaty, Kazakhstan; c Department of Pathology, Asfendiyarov Kazakh National Medical University, Almaty, Kazakhstan; d Department of Diagnostic Radiology, Asfendiyarov Kazakh National Medical University, Almaty, Kazakhstan; e Department of Phthisiology, Asfendiyarov Kazakh National Medical University, Almaty, Kazakhstan; f Kazakh Research Institute of Oncology and Radiology, Almaty, Kazakhstan; g City Clinical Hospital No. 7, Almaty, Kazakhstan

**Keywords:** Sarcoidosis, Neurosarcoidosis, Pulmonary fibrosis, Obstructive hydrocephalus, Magnetic resonance imaging, Computed tomography, Radiologic–pathologic correlation

## Abstract

Sarcoidosis is a systemic granulomatous disease characterized by non-caseating granulomas, most commonly affecting the lungs and intrathoracic lymph nodes. Central nervous system involvement (neurosarcoidosis) is rare but associated with significant morbidity and mortality, particularly in advanced systemic disease. We report a fatal case of a 43-year-old man with long-standing pulmonary sarcoidosis who developed progressive neurosarcoidosis complicated by obstructive hydrocephalus and subsequent ventriculoperitoneal shunt infection. High-resolution computed tomography demonstrated advanced perilymphatic and peribronchovascular fibrosis with symmetric mediastinal lymphadenopathy, consistent with fibrotic pulmonary sarcoidosis (Scadding stage IV). Contrast-enhanced brain magnetic resonance imaging revealed a large, intensely enhancing mass-like lesion centered in the atrium of the right lateral ventricle, with extension into the posterior and temporal horns, causing near-complete obliteration of the ventricular lumen and secondary obstructive hydrocephalus. Autopsy showed extensive fibrotic and granulomatous remodeling of the lungs with non-caseating granulomas. Histological examination of tissue obtained from the central nervous system lesion demonstrated non-necrotizing granulomatous inflammation, supporting central nervous system involvement; however, the precise anatomical origin of the intraventricular lesion was not established microscopically. This case highlights a rare and fatal presentation of sarcoidosis with combined fibrotic pulmonary involvement and neurosarcoidosis. Mechanical obstruction by the mass-like intraventricular lesion played the principal role in the development of hydrocephalus. Radiologic–pathologic correlation provided important insights into the underlying disease mechanisms.

## Introduction

Sarcoidosis is a systemic granulomatous disease of unknown etiology characterized by the formation of non-caseating granulomas, most commonly affecting the lungs and intrathoracic lymph nodes [1–3]. Its prevalence varies widely across geographic and ethnic populations, ranging from 2 to 160 cases per 100,000 individuals, with reported 5-year mortality rates of up to 7–10% [4–6]. In recent decades, an increase in sarcoidosis-related mortality has been reported in several regions, including Japan and Northern Europe, largely attributed to progressive fibrotic pulmonary disease and extrapulmonary involvement [5, 6]. Fibrotic pulmonary sarcoidosis is a major determinant of long-term outcomes. It develops in approximately 10–40% of patients and is characterized by irreversible interstitial remodeling, pulmonary hypertension, and progressive respiratory failure [2, 3, 7, 8]. Recent studies have highlighted the prognostic value of both functional and radiological parameters, including the composite physiologic index, the pulmonary artery-to-aorta diameter ratio, and the extent of fibrosis on high-resolution computed tomography (HRCT) [7, 8]. These parameters reflect not only the extent of fibrotic changes but also vascular involvement, which plays a crucial role in disease progression and mortality.

Extrapulmonary manifestations significantly complicate the clinical course of sarcoidosis. Among these, neurosarcoidosis is one of the most severe yet relatively uncommon forms, occurring in approximately 5–15% of patients and capable of affecting any part of the central or peripheral nervous system [9–13]. It may involve the meninges, brain parenchyma, vasculature, and cerebrospinal fluid pathways, leading to hydrocephalus, focal neurological deficits, seizures, and altered consciousness [11, 14]. Diagnosis remains challenging, as clinical and laboratory findings are often nonspecific. Contrast-enhanced magnetic resonance imaging (MRI) and cerebrospinal fluid analysis play a central role in the diagnostic evaluation.

A particularly severe manifestation of neurosarcoidosis is granulomatous vasculitis, which may lead to ischemic stroke and intracranial hemorrhage [15–17]. These vascular complications provide the structural basis for progressive brain injury and are associated with a markedly worse prognosis. Infectious complications represent an additional major contributor to mortality in sarcoidosis, driven by immune dysregulation and the widespread use of immunosuppressive therapy [18, 19]. Patients with severe and multisystem disease are at particularly high risk of serious infections, which may precipitate systemic decompensation. Despite advances in the understanding of sarcoidosis pathophysiology, the combined presentation of fibrotic pulmonary sarcoidosis and neurosarcoidosis with cerebrospinal fluid obstruction remains poorly characterized, particularly from an integrated radiologic–pathologic perspective. The present case addresses this gap by illustrating the imaging and morphological mechanisms underlying fatal systemic sarcoidosis.

Herein, we present a fatal case of systemic sarcoidosis with advanced fibrotic pulmonary involvement and neurosarcoidosis complicated by obstructive hydrocephalus and infectious complications, with detailed radiologic–pathologic correlation.

## Case Report

### Investigations

#### Radiologic findings

A 43-year-old man had been followed for pulmonary sarcoidosis since 2018 and had received intermittent corticosteroid therapy. Despite treatment, the disease progressed to fibrotic pulmonary sarcoidosis (Scadding stage IV), characterized radiologically by extensive perihilar and peribronchovascular fibrosis with marked architectural distortion on HRCT [2, 3]. In 2024, he developed progressive neurological symptoms, including headache, confusion, and left-sided weakness.

**Figure 1:**
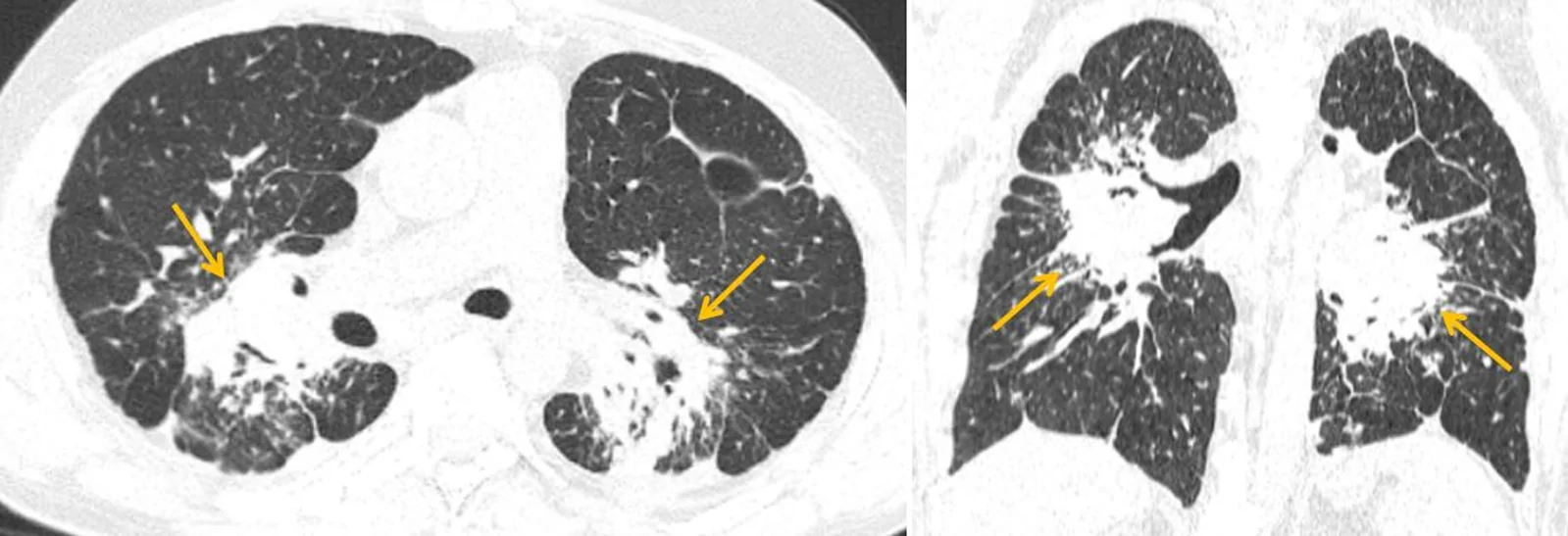
Axial and coronal HRCT images (lung window) demonstrating heterogeneous perihilar consolidation with airless conglomerates (yellow arrows), preserved bronchial patency, and linear pleural adhesions. HRCT: high-resolution computed tomography.

**Figure 2:**
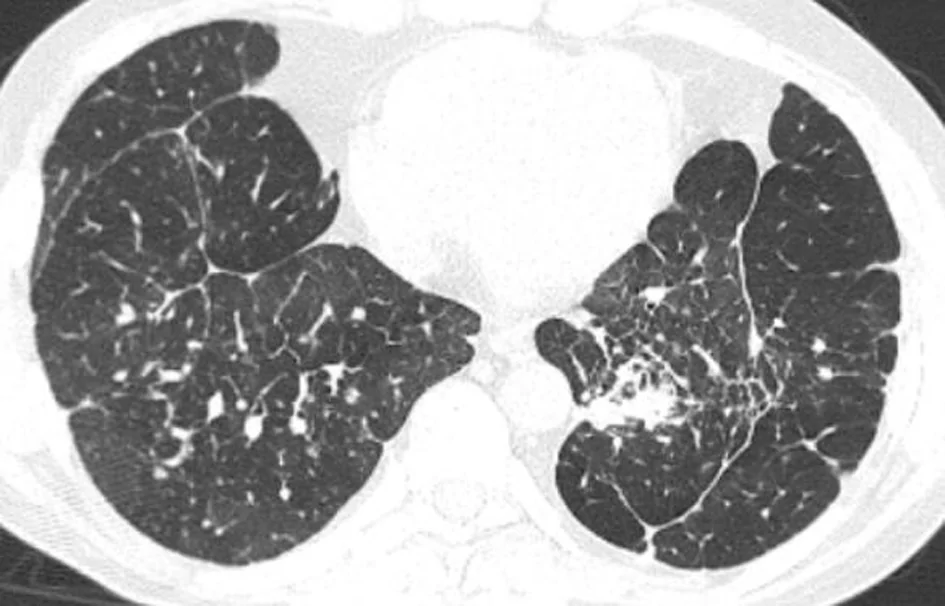
Axial HRCT image (lung window) demonstrating diffuse interstitial reticular changes with interlobular septal thickening, nodular thickening of bronchovascular bundles, and ill-defined ground-glass opacities with preserved bronchial patency. HRCT: high-resolution computed tomography.

**Figure 3:**
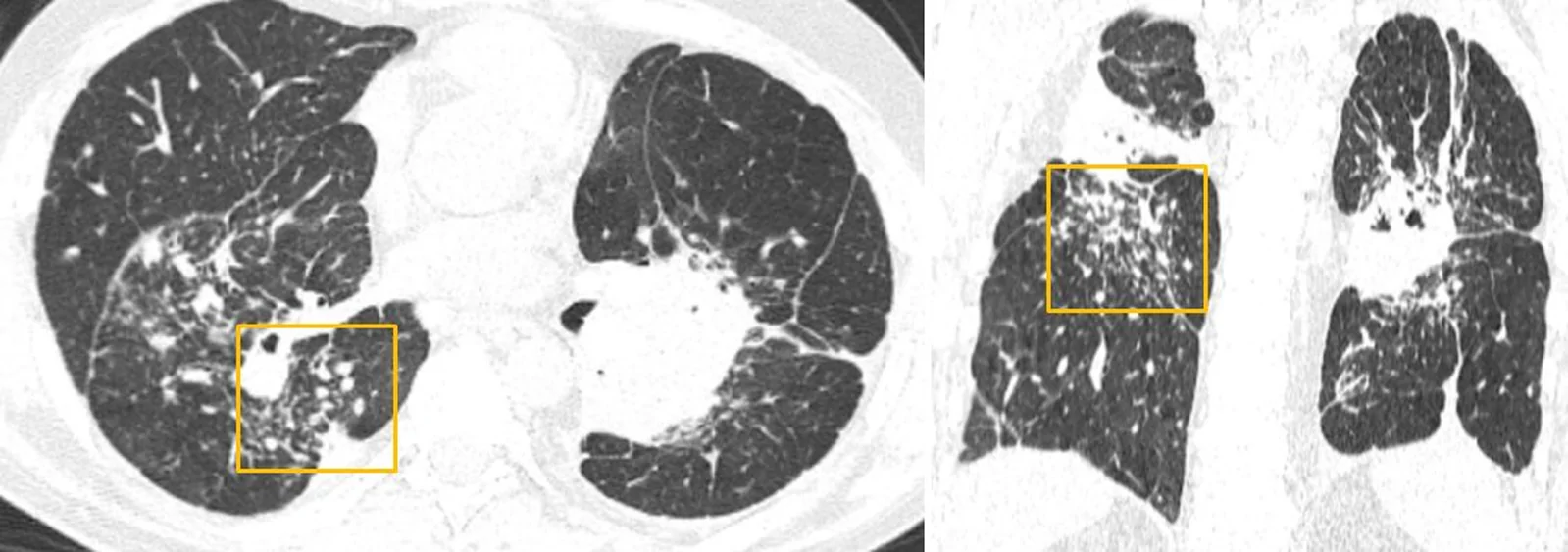
Axial and coronal HRCT images (lung window) demonstrating multiple polymorphic nodules (yellow box) distributed along the bronchovascular bundles and interlobar pleura, without evidence of coalescence. HRCT: high-resolution computed tomography.

**Figure 4:**
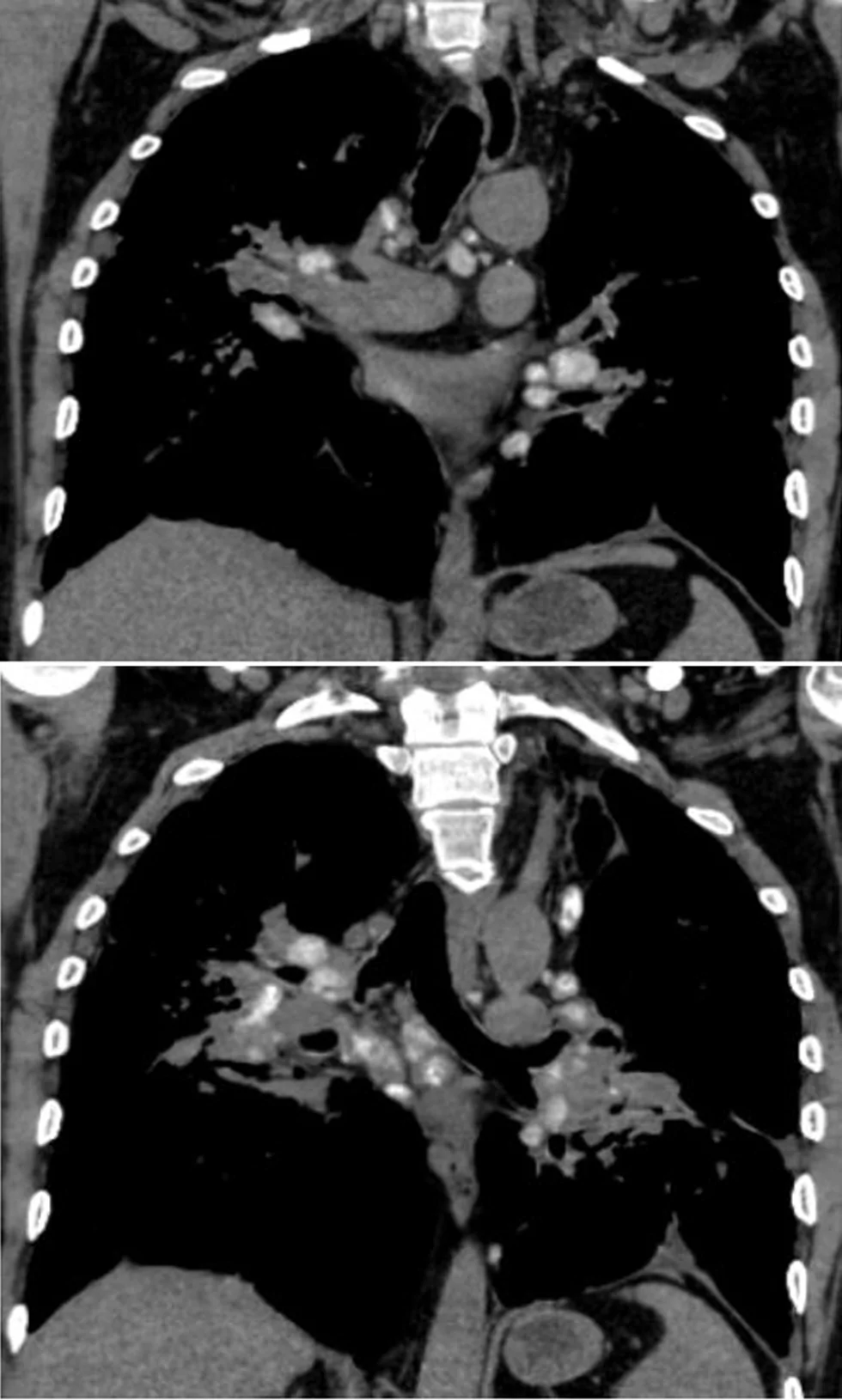
Coronal HRCT image (mediastinal window) demonstrating multiple enlarged mediastinal and hilar lymph nodes involving the upper and lower paratracheal, subaortic, pulmonary, subcarinal, and paraaortic stations. HRCT: high-resolution computed tomography.

Serial HRCT scans demonstrated free and partially loculated pleural effusions along the costal pleura and within the upper portion of the left major interlobar fissure, as well as in the posterior costophrenic recess of the right lung, with maximal thicknesses of 2.5, 1.4, and 1.3 cm, respectively. In the perihilar regions of both lungs, heterogeneous parenchymal consolidations were observed, characterized by airless conglomerates interspersed with areas of preserved patency of lobar, segmental, and subsegmental bronchi, along with linear pleural adhesions (Fig. 1). The remaining lung parenchyma demonstrated marked interstitial reticular changes, including thickening of interlobular and interalveolar septa, polynodular thickening of bronchovascular structures, and ill-defined areas of ground-glass attenuation with preserved bronchial patency (Fig. 2). Multiple polymorphic nodules were distributed along bronchovascular bundles and the interlobar pleura, without evidence of coalescence (Fig. 3). Additionally, multiple enlarged mediastinal and bilateral hilar lymph nodes with well-defined margins were identified in the paratracheal, subaortic, hilar, subcarinal, and paraaortic stations (Fig. 4).

**Figure 5:**
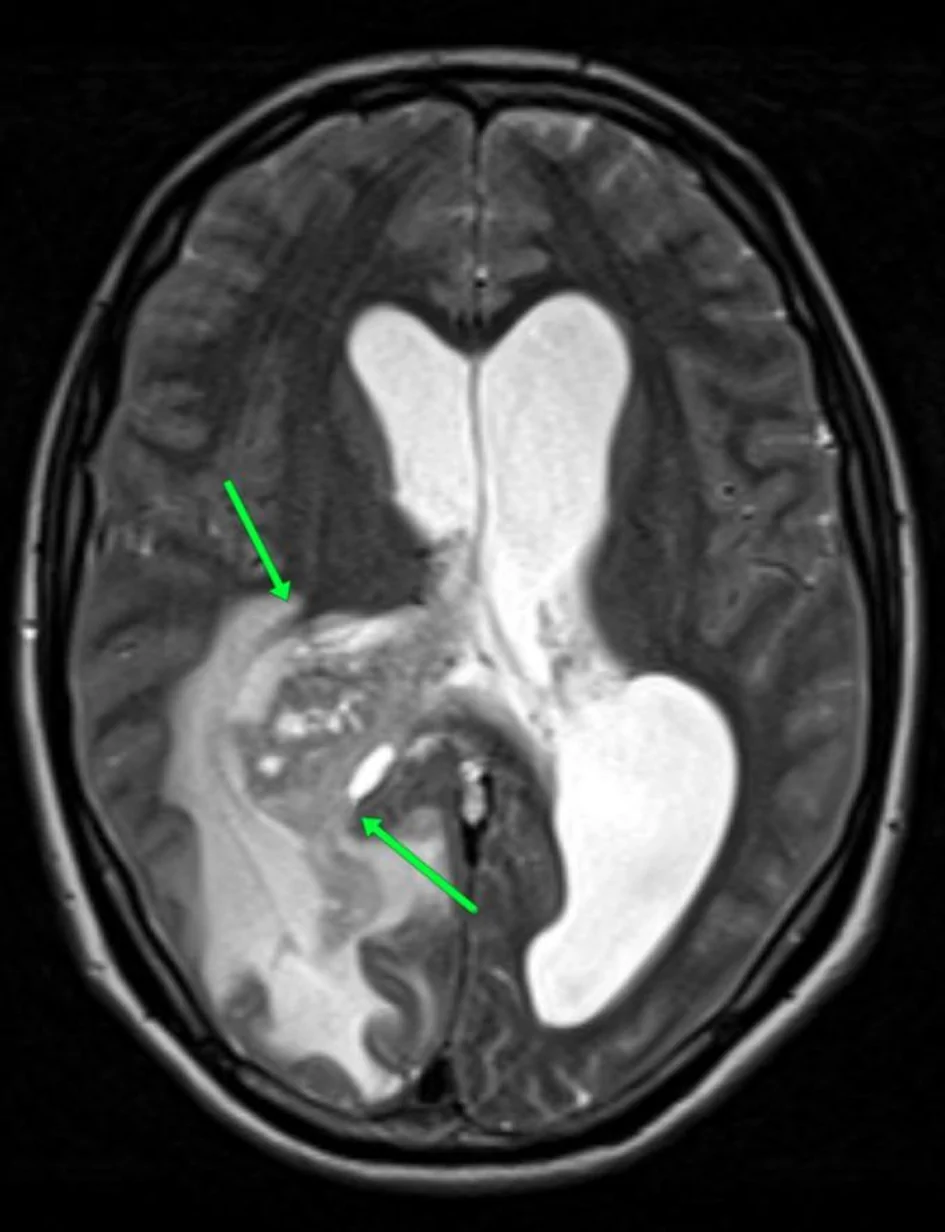
Axial T2-weighted MRI demonstrating a large intraventricular mass (green arrows) arising from the choroid plexus in the atrium of the right lateral ventricle, measuring approximately 6–7 cm in maximal dimension and greater than 3 cm in thickness. MRI: magnetic resonance imaging.

**Figure 6:**
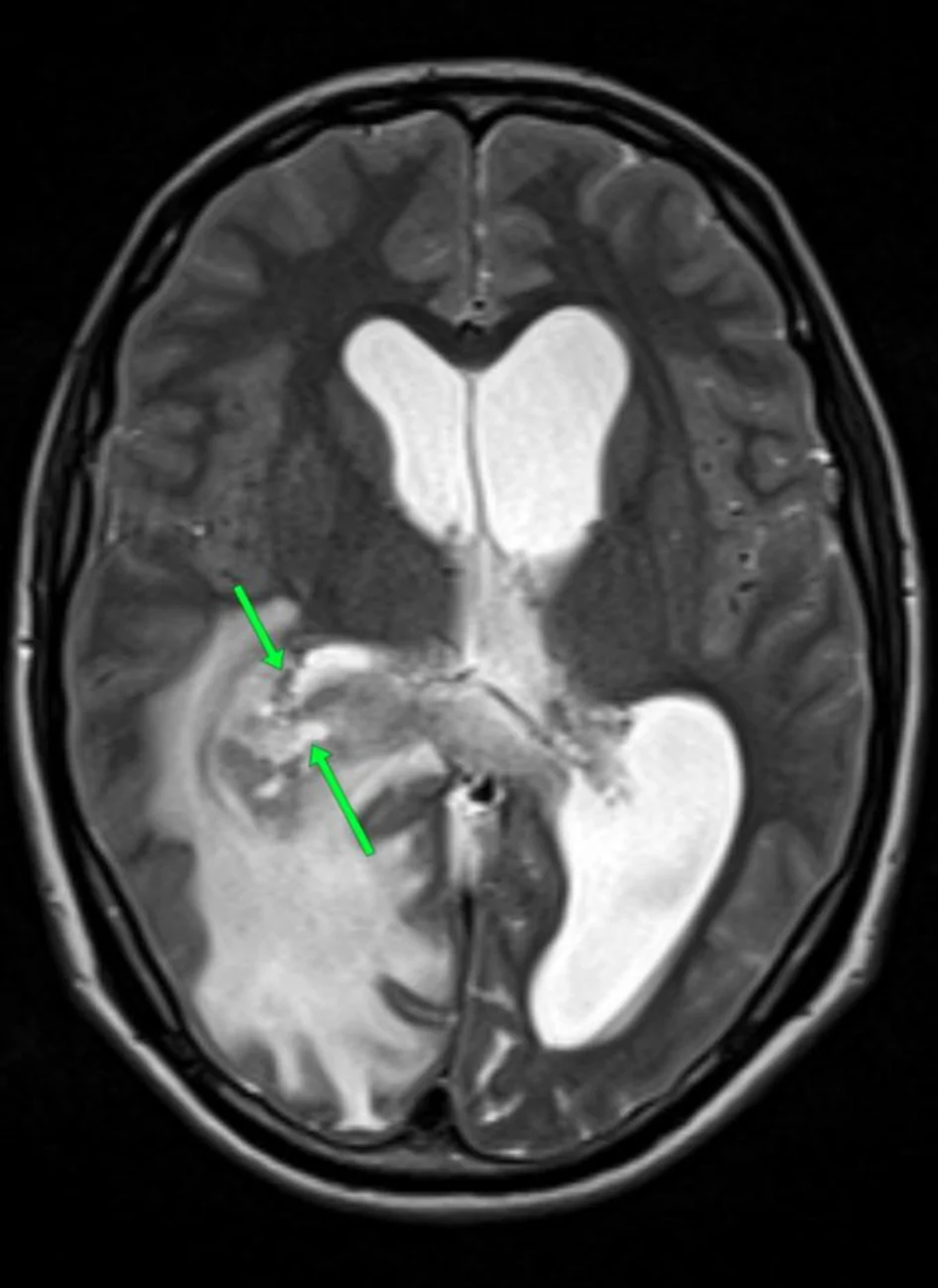
Axial T2-weighted MRI demonstrating a heterogeneous lesion with multiple septations and mixed cystic and solid components, without diffusion restriction on DWI. DWI: diffusion-weighted imaging; MRI: magnetic resonance imaging.

**Figure 7:**
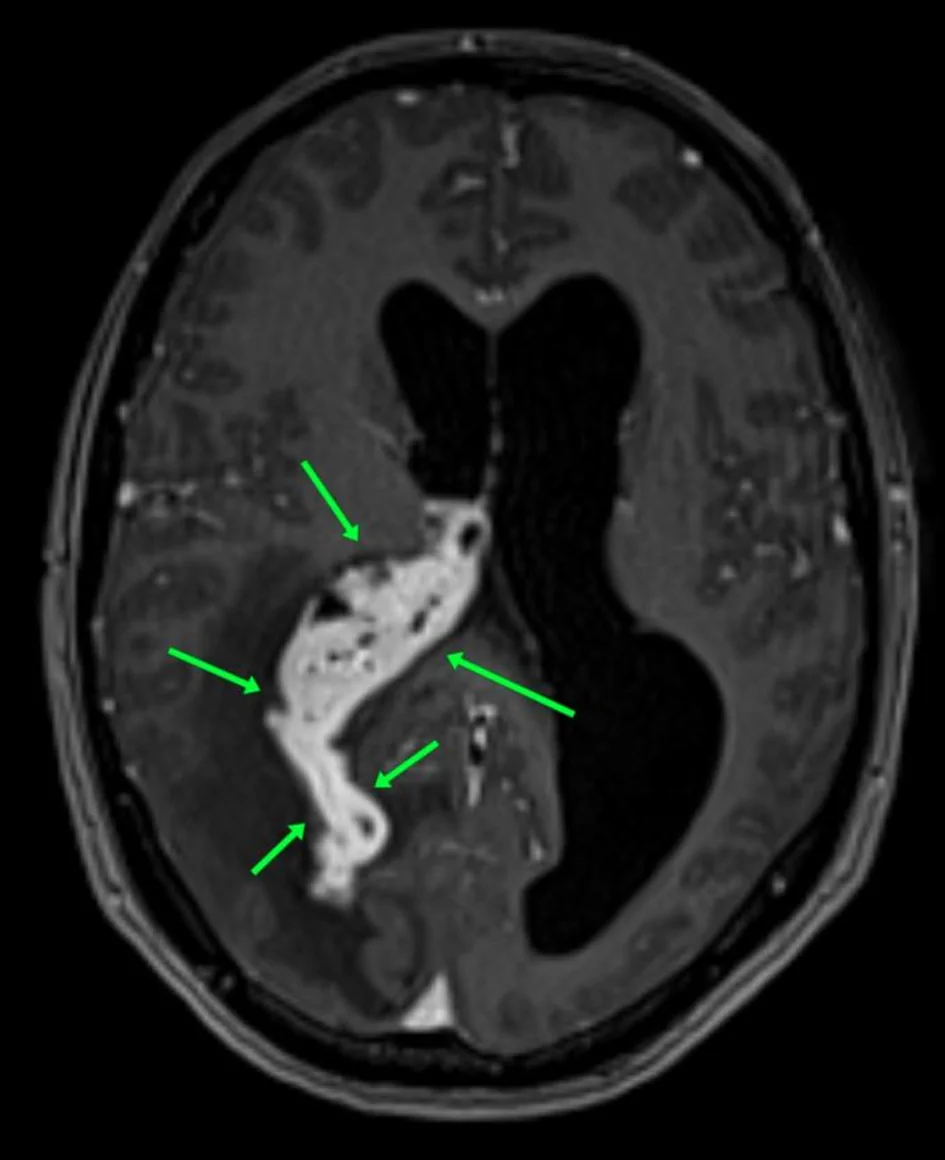
Post-contrast axial T1-weighted MRI demonstrating intense, heterogeneous enhancement of the lesion (green arrows). MRI: magnetic resonance imaging.

**Figure 8:**
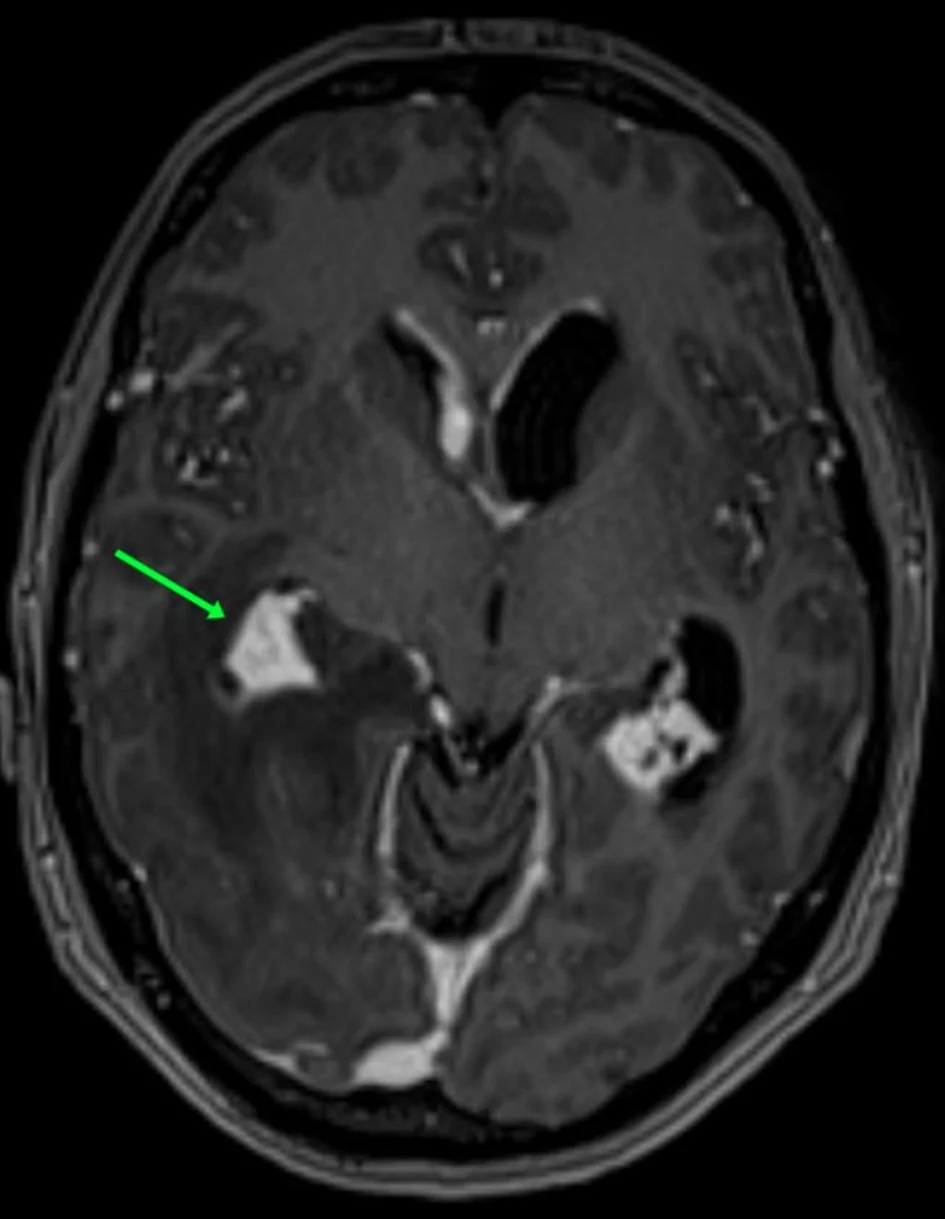
Post-contrast axial T1-weighted MRI demonstrating extension of the lesion into the posterior and temporal horns of the right lateral ventricle, with near-complete obliteration and distortion of the ventricular lumen (green arrows). MRI: magnetic resonance imaging.

**Figure 9:**
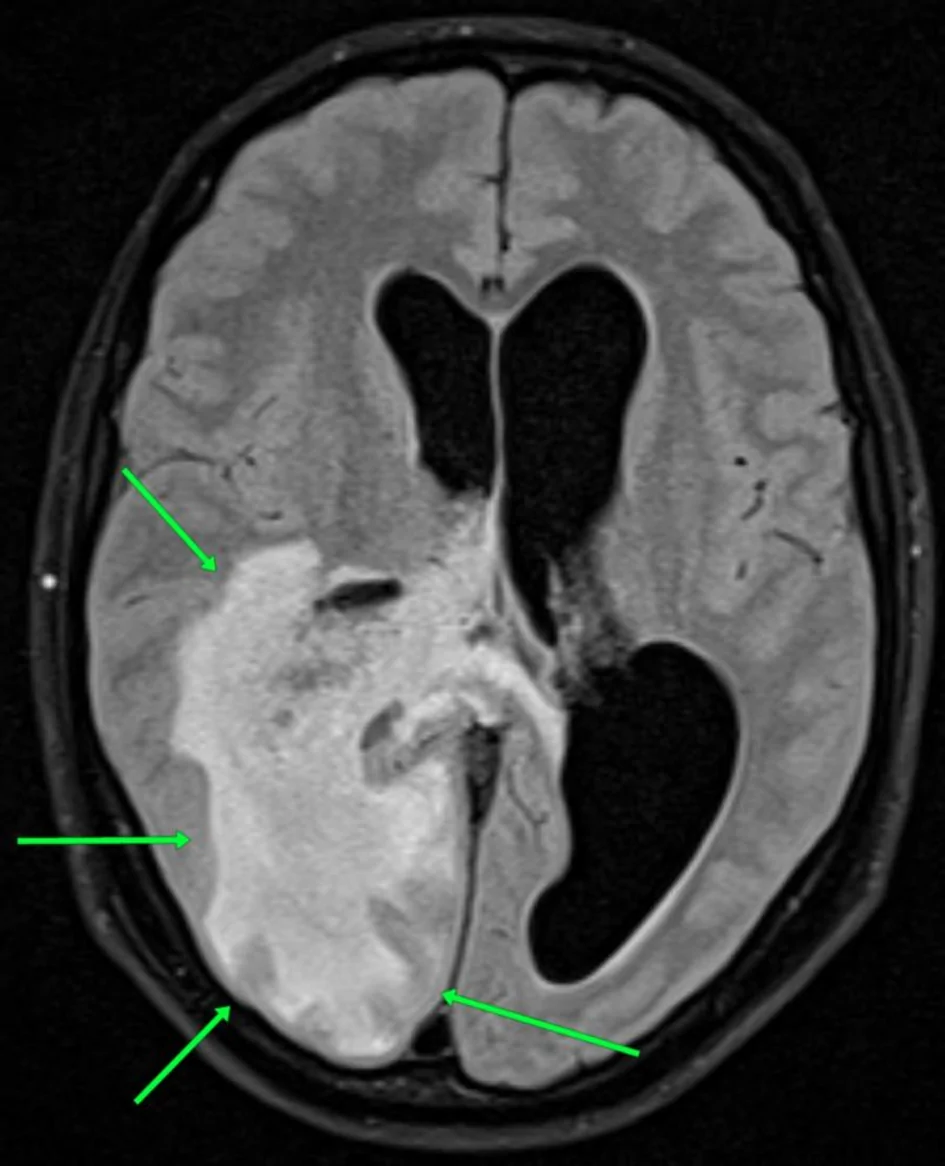
Axial T2-FLAIR MRI demonstrating marked periventricular white matter edema involving the adjacent temporal and occipital lobes (green arrows). FLAIR: fluid-attenuated inversion recovery; MRI: magnetic resonance imaging.

**Figure 10:**
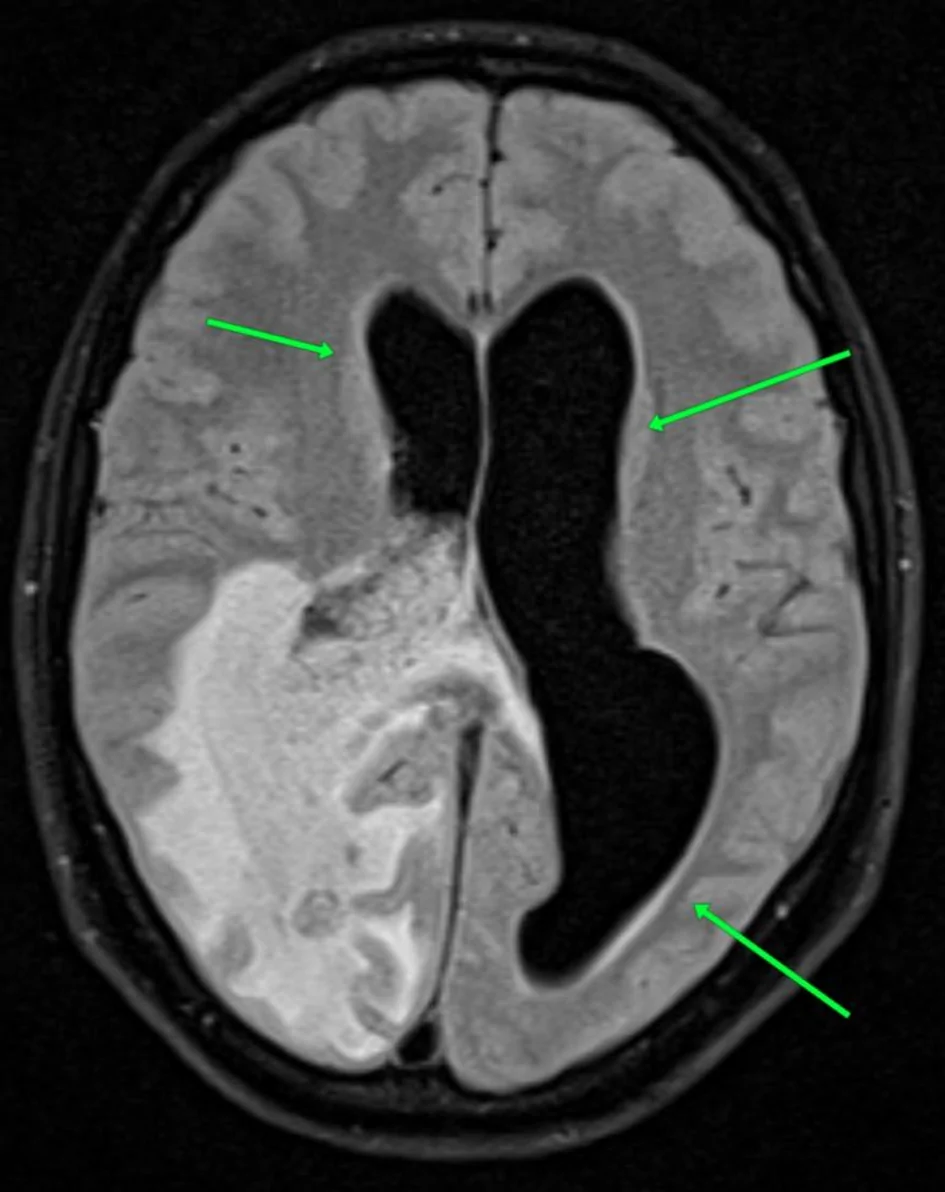
Axial T2-FLAIR MRI demonstrating moderate dilation of the lateral ventricles, more pronounced on the contralateral side (green arrows), consistent with obstructive hydrocephalus. FLAIR: fluid-attenuated inversion recovery; MRI: magnetic resonance imaging.

MRI demonstrated a large mass-like lesion centered in the atrium of the right lateral ventricle and appearing to arise from the choroid plexus, measuring approximately 6–7 cm in maximal dimension and more than 3 cm in thickness (Fig. 5). The lesion exhibited a heterogeneous internal architecture with multiple septations and mixed cystic and solid components, without definite diffusion restriction on diffusion-weighted imaging (Fig. 6). On post-contrast T1-weighted images, the lesion showed intense and heterogeneous enhancement (Fig. 7). It extended into the posterior and temporal horns of the right lateral ventricle, causing marked deformation and near-complete obliteration of their lumina (Fig. 8). Prominent periventricular white matter edema was observed in the adjacent temporal and occipital lobes (Fig. 9). Moderate dilation of the lateral ventricles was present, more pronounced on the contralateral side, consistent with hydrocephalus caused primarily by mechanical obstruction from the large intraventricular mass-like lesion (Fig. 10). The imaging appearance mimicked an intraventricular neoplasm. The postoperative course was complicated by ventriculoperitoneal shunt infection. The patient subsequently died from respiratory failure and cerebral edema.

#### Pathologic findings

**Figure 11:**
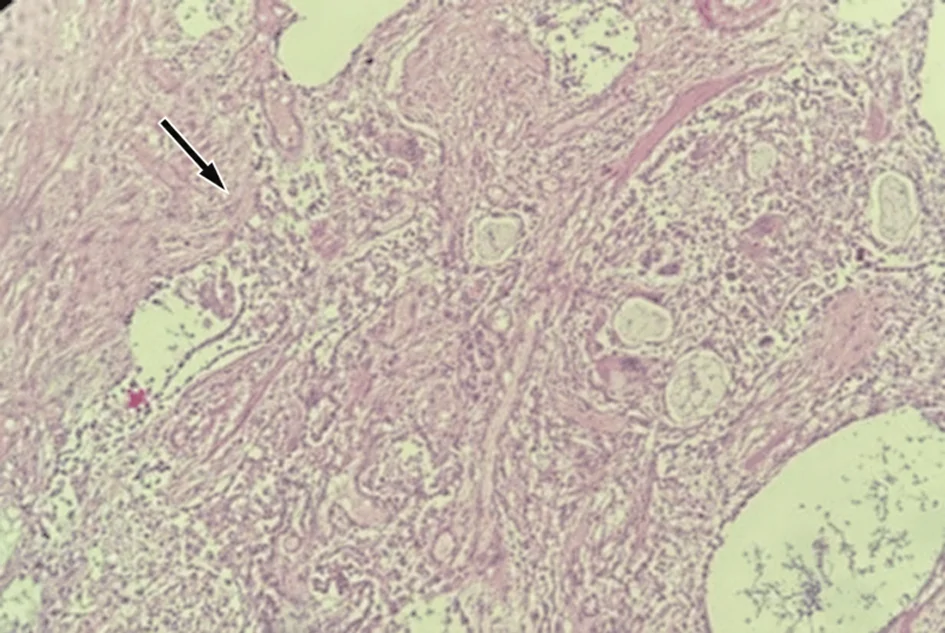
Histological section of the lung demonstrating marked interstitial fibrosis with distortion of the surrounding pulmonary architecture (arrow) and associated chronic inflammatory changes (hematoxylin and eosin stain, × 200).

**Figure 12:**
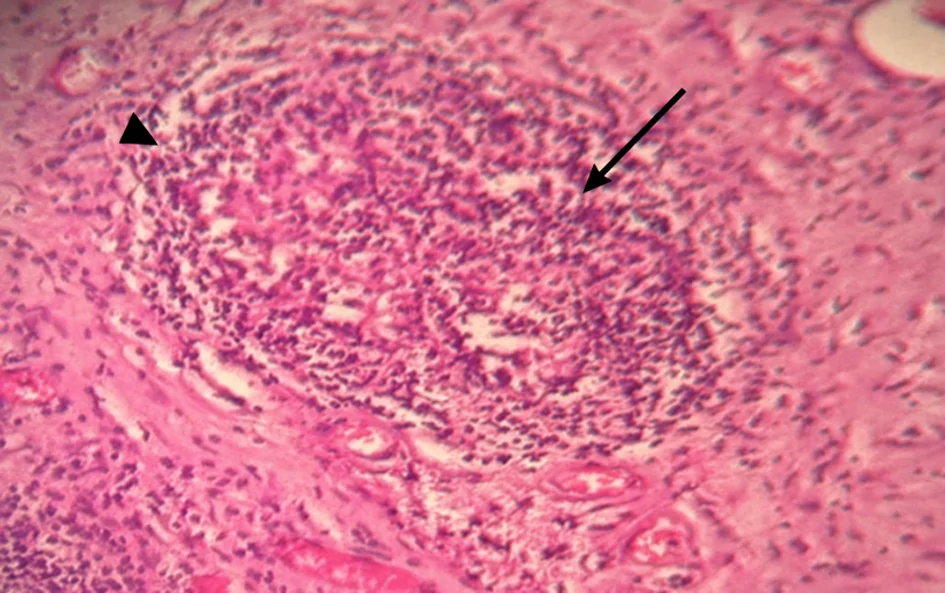
Histological section obtained from a grossly abnormal central nervous system lesion, demonstrating epithelioid granulomatous inflammation (arrow) with a peripheral lymphocytic infiltrate (arrowhead) (hematoxylin and eosin stain, × 200).

**Figure 13:**
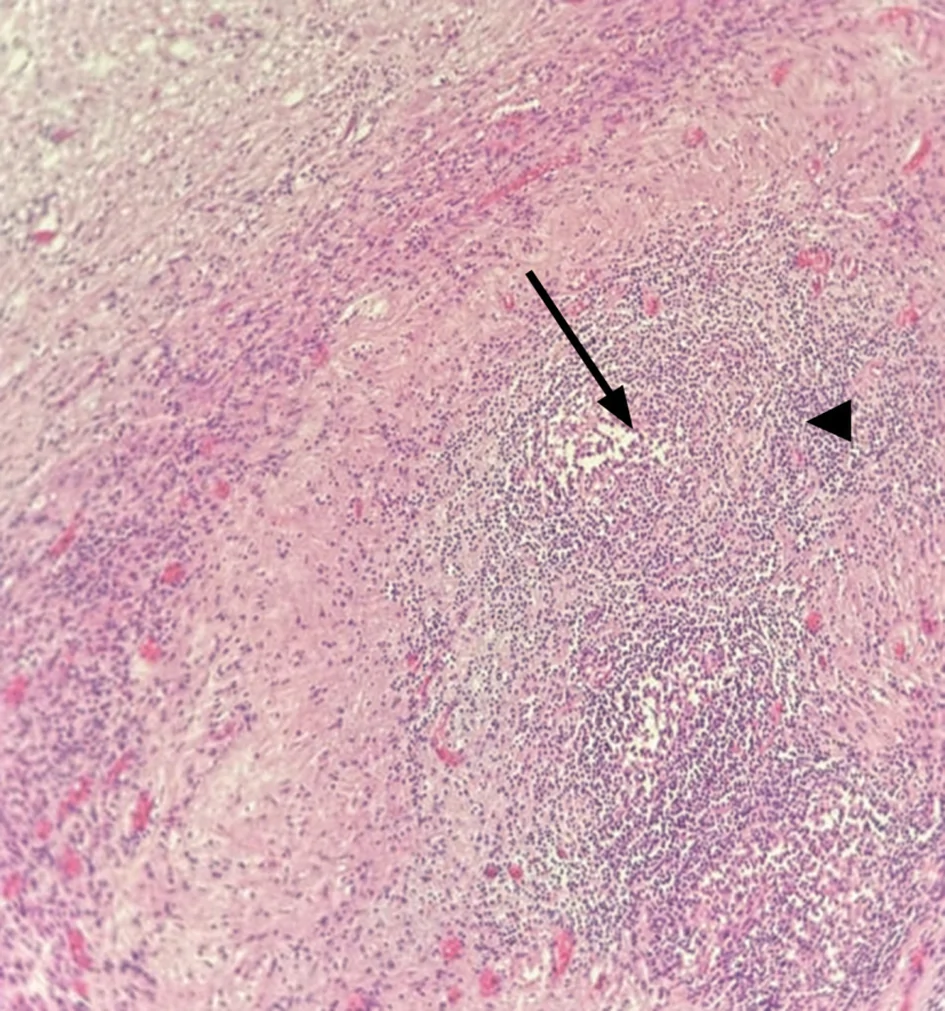
Histological section obtained from the central nervous system lesion demonstrating non-necrotizing granulomatous inflammation (arrow) with a peripheral lymphocytic infiltrate (arrowhead) (hematoxylin and eosin stain, ×200).

**Figure 14:**
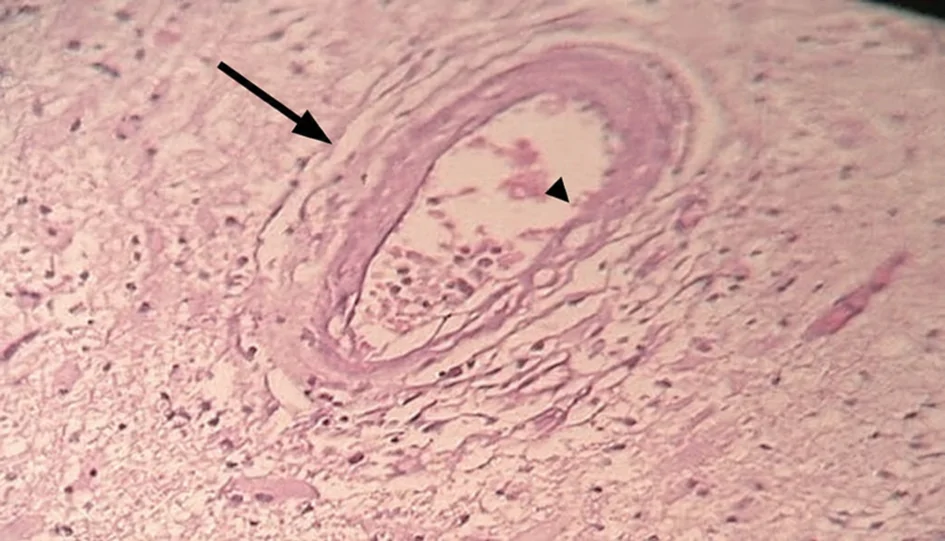
Histological section of a central nervous system vessel demonstrating concentric fibrotic thickening of the vessel wall (arrow) and marked luminal narrowing (arrowhead) (hematoxylin and eosin stain, ×200).

Post-mortem examination included macroscopic assessment of the lungs and brain, followed by histological analysis. Tissue samples for histological examination were obtained from grossly abnormal areas of the brain. The choroid plexus, ventricular ependyma, and periventricular brain parenchyma were examined macroscopically but were not separately sampled for microscopic examination. Consequently, direct histological involvement of these specific anatomical structures could not be assessed. The autopsy and tissue sampling were performed in accordance with the applicable national protocols and recommendations of the Ministry of Health regarding the amount of tissue collected for histological examination. The collected tissue samples were fixed in 10% neutral buffered formalin, embedded in paraffin, sectioned at 5 µm, and stained with hematoxylin and eosin. Additional stains for mycobacteria and fungi, including Ziehl–Neelsen/Fite or auramine, PAS, and Grocott/GMS, as well as tissue cultures, PCR-based tests, and other microbiological studies, were not performed on the pulmonary or central nervous system specimens. Histopathological interpretation was therefore made in conjunction with the previously established biopsy-based diagnosis of pulmonary sarcoidosis and the clinical and radiological findings. Gross examination of the brain revealed a firm, well-demarcated nodular lesion with a fibrotic capsule and central yellowish degeneration, consistent with a sarcoid granuloma involving the central nervous system. Gross examination of the brain also demonstrated ependymal thickening and distortion of the ventricular system. On gross examination, the lungs were dense, airless, and markedly fibrotic, with multiple granulomatous nodules and pronounced traction-related architectural distortion. Histological examination of the lungs demonstrated well-formed non-caseating granulomas within a markedly fibrotic pulmonary interstitium (Fig. 11). Histological examination of tissue obtained from the grossly abnormal central nervous system lesion demonstrated non-necrotizing granulomatous inflammation with a peripheral lymphocytic infiltrate (Figs. 12 and 13). Concentric fibrotic thickening of vascular walls with luminal narrowing was also observed (Fig. 14); however, definite granulomatous inflammation involving the vessel wall was not demonstrated. These findings supported granulomatous involvement of the central nervous system but did not establish the precise anatomical origin of the intraventricular lesion or direct microscopic involvement of the choroid plexus, ependyma, leptomeninges, or periventricular parenchyma.

### Diagnosis

Based on clinical presentation, radiologic findings, and post-mortem examination, the final diagnosis was systemic sarcoidosis with advanced fibrotic pulmonary involvement and neurosarcoidosis complicated by obstructive hydrocephalus.

### Treatment

The patient had received intermittent corticosteroid therapy for pulmonary sarcoidosis since 2018. However, the exact corticosteroid agent, dose, duration, and tapering schedule were not available in the medical records. Information regarding the use of other immunosuppressive therapy was also unavailable. Following the development of progressive neurological symptoms and radiological evidence of obstructive hydrocephalus in 2024, a ventriculoperitoneal shunt was placed to relieve intracranial pressure. The exact date of shunt placement was not available.

### Follow-up and outcomes

The postoperative course was documented as being complicated by a ventriculoperitoneal shunt infection and elevated inflammatory markers. However, the source and results of microbiological cultures, the identity of any causative microorganism, and details of antimicrobial treatment were not available for retrospective review. Despite shunt placement and supportive treatment, the patient developed progressive neurological deterioration, respiratory failure, and cerebral edema and subsequently died. The exact dates of these terminal events were not available.

## Discussion

This case demonstrates a rare and fatal combination of fibrotic pulmonary sarcoidosis and neurosarcoidosis with intraventricular involvement. The perilymphatic and peribronchovascular fibrosis observed on HRCT closely corresponded to the macroscopic and histopathological findings in the lungs, confirming advanced fibrotic remodeling. Neuroimaging demonstrated a large mass-like lesion centered in the atrium of the right lateral ventricle, with extension into the posterior and temporal horns and near-complete obliteration of the ventricular lumen. These findings indicate that hydrocephalus resulted primarily from mechanical obstruction by the intraventricular lesion. Although associated ependymal or periventricular involvement may have been present, diffuse ependymal infiltration was not sufficiently demonstrated to establish it as the principal mechanism of cerebrospinal fluid obstruction. The mass-like appearance and intense heterogeneous enhancement initially mimicked an intraventricular neoplasm, representing an important diagnostic pitfall. Although the lesion appeared to arise from the choroid plexus on MRI, the choroid plexus and adjacent ventricular structures were not separately sampled for microscopic examination. Consequently, the radiological impression of a choroid plexus origin could not be directly confirmed pathologically. Histological examination also demonstrated concentric fibrotic thickening of the vascular walls with marked luminal narrowing, which may have contributed to chronic ischemic injury. However, the available histological section did not demonstrate definite granulomatous inflammation involving the vessel wall and was therefore insufficient to establish granulomatous vasculitis. Infectious complications likely played a significant role in the patient’s clinical deterioration. The ventriculoperitoneal shunt infection may have further aggravated systemic decompensation and contributed to the fatal outcome.

The differential diagnosis of a large, intensely enhancing intraventricular mass centered in the atrium of the lateral ventricle and appearing to arise from the choroid plexus includes choroid plexus papilloma, atypical choroid plexus papilloma, choroid plexus carcinoma, intraventricular meningioma, ependymal neoplasms, primary central nervous system lymphoma, and metastatic disease. Infectious and inflammatory mimickers include intraventricular tuberculoma, fungal granuloma, inflammatory pseudotumor, foreign-body granulomatous reaction, and other noninfectious granulomatous disorders. Rare granulomatous lesions of the choroid plexus caused by neurosarcoidosis or tuberculosis may closely mimic an intraventricular neoplasm [20, 21]. In the present case, the choroid plexus–centered location, heterogeneous internal architecture, and intense enhancement were not specific and initially raised concern for a neoplasm. The absence of definite diffusion restriction made a highly cellular lesion such as lymphoma less typical but did not exclude it. Identification of non-necrotizing granulomatous inflammation supported a granulomatous process, and the final interpretation required integration of the clinical history, imaging findings, and pathological examination.

The Neurosarcoidosis Consortium Consensus Group criteria emphasize a compatible neurological presentation and diagnostic evaluation, pathological evidence of systemic or neural sarcoidosis, and rigorous exclusion of alternative causes [22]. In this patient, neurosarcoidosis was supported by the previously established biopsy-based diagnosis of pulmonary sarcoidosis, progressive neurological symptoms, the mass-like intraventricular lesion with obstructive hydrocephalus on MRI, and non-necrotizing granulomatous inflammation in tissue obtained from the central nervous system lesion. Nevertheless, alternative infectious and foreign-body granulomatous conditions could not be definitively excluded. Accordingly, the diagnosis was based on the combined clinical, radiological, and pathological findings and should be regarded as strongly supported rather than independently proven by routine hematoxylin and eosin examination alone.

Treatment of neurosarcoidosis should be individualized according to the neurological phenotype, disease severity, systemic involvement, and risk of irreversible neurological injury. Glucocorticoids remain the usual initial treatment, whereas severe central nervous system phenotypes may warrant the early addition of a steroid-sparing immunosuppressive agent. Methotrexate, azathioprine, and mycophenolate mofetil are commonly used, while tumor necrosis factor-αinhibition, particularly with infliximab, may be considered in refractory disease or selected phenotypes associated with a poor prognosis [23, 24]. Hydrocephalus caused by mechanical obstruction may require urgent cerebrospinal fluid diversion in addition to anti-inflammatory treatment. In the present case, ventriculoperitoneal shunting addressed the ventricular obstruction; however, the exact corticosteroid regimen and information regarding other immunosuppressive therapy were unavailable, precluding a detailed assessment of the treatment response. The subsequent shunt infection also illustrates the importance of balancing immunosuppressive treatment against the risk of infectious complications and closely monitoring neurosurgical devices.

Such a combined presentation of fibrotic pulmonary sarcoidosis and neurosarcoidosis with obstructive hydrocephalus is exceedingly rare and has been reported only sporadically in the literature. This case highlights the importance of early recognition of central nervous system involvement in patients with advanced sarcoidosis and underscores the critical role of contrast-enhanced MRI in detecting potentially life-threatening complications.

This case has several limitations. First, special stains for mycobacteria and fungi, including Ziehl–Neelsen/Fite or auramine, periodic acid–Schiff, and Grocott–Gomori’s methenamine silver, as well as microbiological cultures and polymerase chain reaction-based studies, were not performed on the pulmonary or central nervous system autopsy specimens. Therefore, concomitant infectious granulomatous disease could not be definitively excluded in the autopsy material. Second, immunohistochemical characterization of the granulomatous infiltrates and examination under polarized light for foreign material were not performed, and serum angiotensin-converting enzyme levels were not available. Third, the absence of separate microscopic sampling of the choroid plexus, ventricular ependyma, and periventricular parenchyma limited the site-specific gross-to-microscopic correlation and prevented pathological confirmation of the precise anatomical origin of the intraventricular lesion. Fourth, detailed clinical documentation regarding the corticosteroid regimen, other immunosuppressive therapy, the exact timing of neurological deterioration and shunt placement, microbiological findings, and antimicrobial treatment was unavailable, limiting precise reconstruction of the clinical timeline. Fifth, additional high-quality low- and high-power photomicrographs were not available, limiting the simultaneous assessment of the overall architecture and cellular composition of the granulomatous lesions and their precise anatomical relationship to the ventricular structures. Nevertheless, pulmonary sarcoidosis had previously been established by histological and histochemical examination of a pleural biopsy and confirmed during independent external pathological review. Accordingly, the autopsy findings were interpreted in conjunction with the previously established diagnosis and the overall clinical, radiological, and pathological findings.

### Conclusion

Systemic sarcoidosis may progress to a devastating combination of fibrotic pulmonary disease and neurosarcoidosis. Integrated HRCT, MRI, and pathological correlation enable precise identification of the underlying disease mechanisms, including lymphatic-centered pulmonary fibrosis and mass-like intraventricular involvement of the central nervous system resulting in mechanical cerebrospinal fluid obstruction, which ultimately determine prognosis and outcome. Early recognition of central nervous system involvement and the use of contrast-enhanced imaging are critical for the timely diagnosis of life-threatening complications.

### Learning points

Neurosarcoidosis may rarely present as a large mass-like intraventricular lesion mimicking a neoplasm. In this case, obstructive hydrocephalus was caused primarily by mechanical obstruction of the ventricular lumen by the intraventricular lesion. Contrast-enhanced MRI is essential for characterizing active central nervous system involvement, as non-contrast imaging may underestimate the extent of disease. The coexistence of fibrotic pulmonary sarcoidosis and neurosarcoidosis defines a severe multisystem phenotype associated with poor prognosis. Radiologic–pathologic correlation is important for accurate diagnosis and for understanding the underlying disease mechanisms.

## Data Availability

The authors declare that the data supporting the findings of this study are available within the article.
